# Novel Compound Missense and Intronic Splicing Mutation in *ALDH18A1* Causes Autosomal Recessive Spastic Paraplegia

**DOI:** 10.3389/fneur.2021.627531

**Published:** 2021-05-19

**Authors:** Yi-Jun Chen, Zai-Qiang Zhang, Meng-Wen Wang, Yu-Sen Qiu, Ru-Ying Yuan, En-Lin Dong, Zhe Zhao, Hai-Tao Zhou, Ning Wang, Wan-Jin Chen, Xiang Lin

**Affiliations:** ^1^Department of Neurology and Institute of Neurology, The First Affiliated Hospital of Fujian Medical University, Fuzhou, China; ^2^Department of Neurology, Beijing Tiantan Hospital, Capital Medical University, Beijing, China; ^3^Department of Neuromuscular Disorders, The Third Hospital of Hebei Medical University, Shijiazhuang, China; ^4^Department of Neurology, Luoyang Central Hospital Affiliated to Zhengzhou University, Luoyang, China; ^5^Fujian Key Laboratory of Molecular Neurology, Fujian Medical University, Fuzhou, China

**Keywords:** spastic paraplegia 9, *ALDH18A1*, intronic splicing mutation, SpliceAI, RNA splicing assay

## Abstract

**Background:** Hereditary spastic paraplegia (HSP) caused by mutations in *ALDH18A1* have been reported as spastic paraplegia 9 (SPG9), with autosomal dominant and autosomal recessive transmission (SPG9A and SPG9B). SPG9 is rare and has shown phenotypic and genotypic heterogeneity in previous reports.

**Methods:** This study screened *ALDH18A1* mutations in autosomal recessive HSP patients using combined whole exome sequencing and RNA splicing analysis. We conducted *in silico* investigations, co-segregation analysis, and ELISA-based analysis of P5CS (Δ1-pyrroline-5-carboxylate synthetase; encoded by *ALDH18A1*) concentration to validate the pathogenicity of the detected *ALDH18A1* variants. All previously reported bi-allelic *ALDH18A1* mutations and cases were reviewed to summarize the genetic and clinical features of *ALDH18A1*-related HSP.

**Results:** A novel missense mutation c.880T>C, p.S294P and an intronic splicing mutation c.-28-13A>G were both detected in *ALDH18A1* in an autosomal recessive family presenting with a complicated form HSP. ELISA assays revealed significantly decreased P5CS concentration in the proband's plasma compared with that in the healthy controls. Moreover, review of previously reported recessive cases showed that SPG9B patients in our cohort presented with milder symptoms, i.e., later age at onset and without cognitive impairment.

**Conclusion:** The present study expands the genetic and clinical spectrum of SPG9B caused by *ALDH18A1* mutation. Our work defines new genetic variants to facilitate future diagnoses, in addition to demonstrating the highly informative value of splicing mutation prediction in the characterization of disease-related intronic variants.

## Introduction

Hereditary spastic paraplegia (HSP) is an umbrella group comprised of neurodegenerative diseases characterized by progressive weakness and spasticity of the lower extremities (1). HSP caused by mutations in *ALDH18A1* (MIM#616586), encoding delta-1-pyrroline-5-carboxylate synthase (P5CS), has been reported as hereditary spastic paraplegia type 9 (SPG9), i.e., SPG9A and SPG9B, which are distinguished by autosomal dominant and autosomal recessive modes of inheritance, respectively (2, 3). The P5CS enzyme is known to catalyze proline and ornithine biosynthesis, and disease-related mutations in *ALDH18A1* have been correlated with loss or decrease of P5CS function (4). Patients with SPG9 present with either a pure or complicated form of HSP (3). Pure forms are characterized by lower limb spasticity, without prominent additional clinical findings other than mild urinary symptoms and impaired distal vibratory sensation (5). Complicated forms are associated with additional, often severe, clinical features such as cognitive impairment, seizures, neuropathy, amyotrophy, short stature, and vision abnormalities, among others (6).

HSP exhibits considerable genetic heterogeneity with more than 80 different HSP (or SPG) loci reported, including *ALDH18A1* (2, 7). Massively parallel high throughput sequencing strategies, such as targeted gene-panel sequencing, whole exome sequencing (WES), and whole genome sequencing (WGS), have facilitated genetic diagnosis of HSP (8–11). As a first-line test, WES can significantly reduce the time and cost of the diagnostic process through accelerated identification of the causative variants associated with HSP. However, the rate of successful diagnoses may only reach ~50% in carefully selected cohorts of patients (12, 13). Unbiased genome-wide association studies have revealed that non-coding regions can play a significant role in gene regulation, and may account for 90% of causal disease loci in human complicated diseases (13–15). Penetrant non-coding variants that disrupt the normal mRNA splicing patterns, despite lying outside the canonical GC-AG splice sites, have long been recognized to play a significant role in rare genetic diseases (16). However, intronic splice mutations are often overlooked in WES sequence data due to the difficulty in accurate identification of these splice-altering variants outside the canonical GT-AG splice sites.

In this study, we report the identification of a novel splicing mutation c.-28-13A>G in intron 1 and a missense mutation c.880T>C, p.S294P in the *ALDH18A1* gene in an autosomal recessive family presenting with a complicated form HSP. This is the first time that a hidden intronic mutation has been described for SPG9B. The mutation disrupts the normal accepter site, leading to the creation of an aberrant transcript that lacks exon 2, in which the original, correct AUG start codon is located. Thus, internal translation may initiate at downstream AUG start sites, thus leading to hypomorphic protein, and potentially resulting in mRNA degradation due to non-sense-mediated mRNA decay. Our results demonstrate that the genetic causes of Mendelian-inheritance diseases can be determined by focusing on non-coding DNA sequences.

## Materials and Methods

### Patients and Samples

The proband was recruited and examined at the Department of Neurology of the First Affiliated Hospital of Fujian Medical University. Detailed neurological examinations were performed on all members of the family by at least two senior neurologists. Blood samples were collected according to standard methods. Written informed consent was obtained from all participants in this study. The research project was approved by the Ethics Committee of the First Affiliated Hospital of Fujian Medical University.

### Whole Exome Sequencing

Genomic DNA was extracted from the peripheral blood cells of patients and normal controls using Qiagen kits. Whole exome DNA was captured using a SureSelect Human All Exon V6 kit (Agilent) and sequenced using the Illumina HiSeq 3000 platform as described in our previous study (17). Fragment sequences were aligned to the consensus sequence (UCSC hg38). Variant calling was performed by Genome Analysis Toolkit (GATK) (18) and annotated with ANNOVAR (19). We filtered the SNP sets obtained from the total sequencing data using the parameters of GQ>20 and DP>8 for genotype quality (GQ) and read depth (DP), respectively. Variants were excluded based on the following criteria: (a) the variants did not affect the amino acid sequence; (b) the allele frequency was >1% in the 1,000 Genomes Project, ESP database, or gnomAD. Then we screened potential disease-causing genes according to their patterns of inheritance.

### RNA Splicing Analysis

Total RNA was isolated from the peripheral blood leukocytes of family members. RNA was extracted using a Trizol extraction kit (Invitrogen, Carlsbad, CA, USA) and then synthesized to cDNA with a PrimeScript RT reagent kit (TAKARA BIO, Kusatsu, Shiga, Japan) according to the manufacturer's protocols. Primers were designed to target *ALDH18A1* exons 1–3 to confirm aberrant splicing (forward:5′-ACGGAAGAAAAAAGAGAGTGAG-3′; reverse: 5′-AAGGACTTGCCATGTGTACG-3′). The products amplified by the above primers were separated by agarose gel electrophoresis. The DNA fragments were then purified from the gels and sent for Sanger sequencing.

### High-Throughput Sequencing

The products amplified by the above primers were used for high-throughput sequencing (HTS). The detailed methods were described in the previous literature (20). In brief, the sequencing library was constructed (New England Biolabs), quality checked, and sequenced with paired-end 150-bp reads using the Illumina HiSeq X-Ten platform (Novogene). Paired end reads of 150 bp were pre-processed to remove adaptors and low quality reads using fastp (v 0.19.6) (21). The following criteria were used to remove the low-quality reads: (i) reads containing more than 15 N bases, (ii) a quality score of < 20, and (iii) more than 50% of the sequenced bases have low quality scores. Clean reads were subsequently aligned to the appropriate reference sequence using the Smith-Waterman algorithm. Mutation types were extracted from the aligned results and the number of all types of mutations were also counted. Subsequently, percentage of diverse transcripts was calculated as the number of reads align to mutant sequence over the total number of reads covering the same locus.

### Enzyme-Linked Immunosorbent Assay

The blood samples from the patient carrying *ALDH18A1* mutations and three gender matched healthy controls were centrifuged and the plasma was fractionated prior to storage at −80°C. The concentration of P5CS, encoded by *ALDH18A1*, was measured by human enzyme linked immunosorbent assay (ELISA) (Shanghai Jianglai Biotech, JL15386). The principle of ELISA kit was based on a double-antibody sandwich enzyme-linked immunosorbent assay to measure the concentration of ALDH18A1 by one step. Briefly, the plasma and the horseradish peroxidase (HRP)-labeled detection antibody were added to the microwells, which were pre-coated with ALDH18A1 antibody. Then, the mixture was incubate at 37°C for an hour. After washing with 1 × PBS, 3,3',5,5'-Tetramethylbenzidine was added as the chromogenic substrate. The wavelength at 450 nm excitation was used to assess the concentration of ALDH18A1 in the subject samples. The exact values were determined to the standard curve.

### Statistical Analysis

Data are presented as mean ± SEM. One-way ANOVA with Dunnett's multiple comparisons test was used and probability values *P* < 0.05 were considered statistically significant. The statistical analyses were performed with GraphPad Prism 5.0.

## Results

### Clinical Description

**Figure 1:**
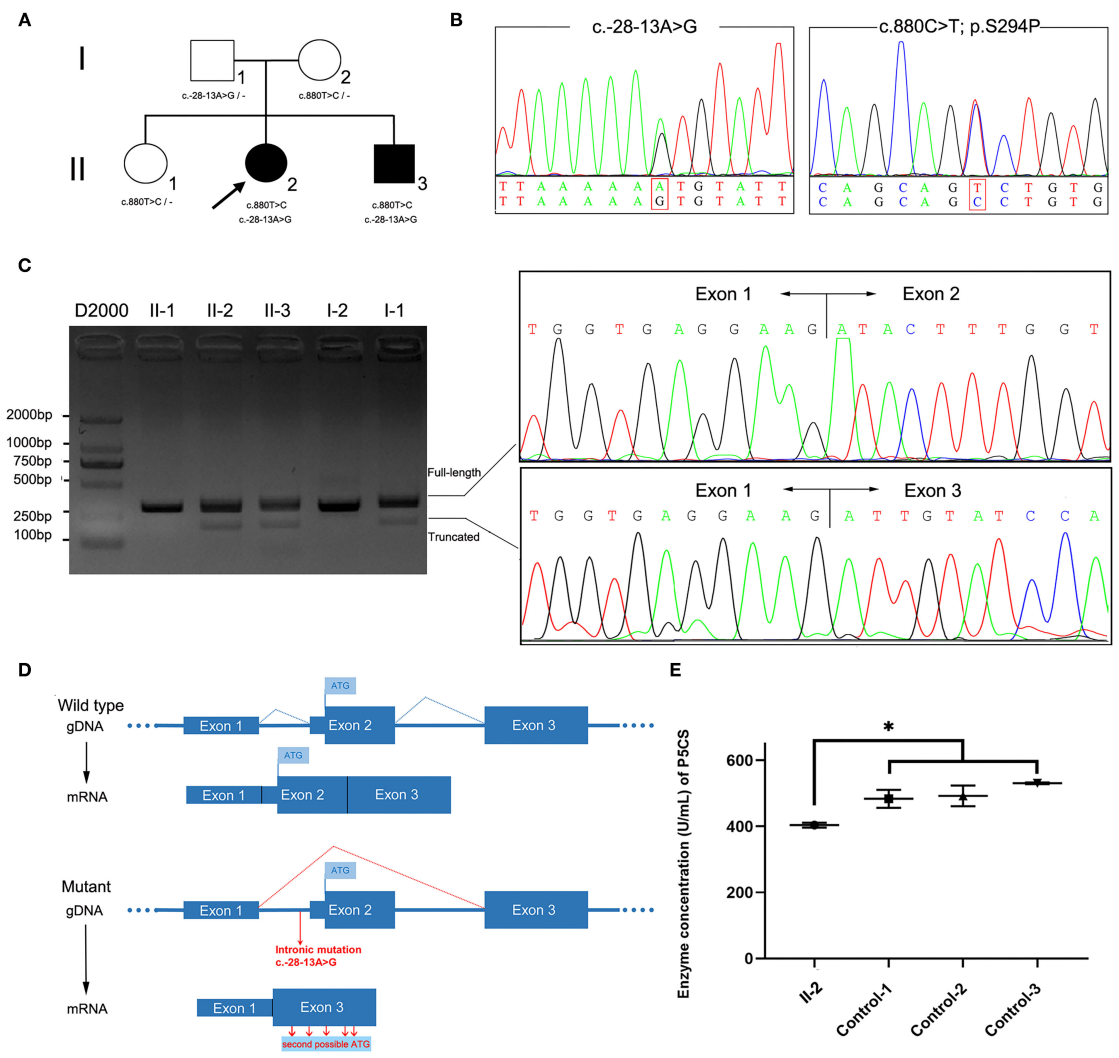
*ALDH18A1* variants in the family and molecular studies. **(A)** Family pedigree and genotype data for the *ALDH18A1* variants. Filled black squares and circles indicate affected individuals. The proband in the family is indicated by an arrow. The genotypes of all available family members were determined. Dash symbol indicates reference allele. **(B)** Sequence analysis of the patients' genomic DNA revealed a splice site variant c.-28-13A>G in intron1 and a missense mutation c.880T>C. **(C)** Agarose gel electrophoresis of RT-PCR products showed an additional fragment (193 bp) in patients II-2 and II-3, but not in healthy family members. Sanger sequencing showed two populations of mRNA, one corresponding to skipped transcription of exon 2. **(D)** Transcriptional consequences of the c.-28-13A> G variant. The schematic diagram above shows the structure of the wild-type *ALDH18A1* transcript (exons 1–3). The following schematic diagram shows the structure of the *ALDH18A1* transcript isoform generated by the c.-28-13A> G variant (exon 2 skipping). **(E)** The plasma P5CS concentration of the proband decreased as compared with that of three, gender matched, healthy controls. Data represent mean ± SEM, ^*^p < 0.05.

The patient II-2 was a 46-year-old woman in ongoing follow-up at the neurology outpatient clinic of the First Affiliated Hospital of Fujian Medical University due to a 20-year-long history of altered gait (Table 1). She was born in a non-consanguineous family and showed normal motor and psychological milestones. She experienced unstable walking and abnormal walking posture at the age of 26 and symptoms worsened after pregnancy. As gait abnormality progressed, she developed dysarthria, although no muscular atrophy or autonomic symptoms were present. No symptoms of cutis laxa were observed. Physical examination revealed decreased muscle strength and increased muscle tone of the lower limbs, hyperreflexia in all limbs, and bilaterally extensor plantar responses. Hoffmann sign was observed in her upper limbs. Spinal MRI presented atrophy of thoracic spinal cord (Supplementary Figure 1). Moreover, the plasma levels of amino acids, including proline, arginine, ornithine, and citrulline, were within the normal range (Supplementary Table 1). The proband had a younger brother, subject II-3, who carried the same mutations. He presented with difficulty in walking in his 30's. Walking abnormalities progressed gradually, but no other symptoms emerged. He can still walk independently at the age of 44. Their parents and elder sister carried the heterozygous mutation without detectable symptoms (Figure 1A).

Table 1Clinical features of patients with bi-allelic mutations in *ALDH18A1* gene.

**Table:** 

**Family N^**°**^/origin**	**This study/Chinese**		**FSP856/Spain** **(****2****)**		**SR45/Portugal** **(****2****)**			
**ALDH18A1 variants**	**c.-28-13A>G; c.880C>T, p.S294P**		**c.2143G>C, p.D715H (hom)**		**c.383G>A, p.R128H;c.1910T>C, p.L637P**			
Individual N° (sex)	F	M	18 (M)	19 (M)	II:3 (F)	II:4 (F)	II:6 (M)	II:9 (F)
Age at onset (years)	26	30	7	7	<1	<1	<1	<1
Symptoms at onset	Spastic gait	Spastic gait	Toe walking, ID	ID, gait difficulties	MD, GR	MD, GR	PMD	Severe MD, GR
Disease duration (years)	20	13	35	32	44	49	46	43
Spasticity at gait	+	+	+	+	+	+	+	+
Weakness	+	+	+	–	+	+	+	+
Increased reflexes LL	+	+	+	+	+	+	+	–
Increased reflexes UL	–	–	+	+	+	+	+	–
Extensor plantar response	+	+	+	+	+	+	+	–
Hoffman sign	–	left+	NA	NA	NA	NA	NA	NA
Decreased vibration sense at ankles	NA	NA	+	+	NA	NA	–	NA
Urinary symptoms	–	–	+	–	+	+	+	+
Cerebellar signs	Dysarthria	–	Postural tremor	Postural tremor	–	–	–	NA
Cognitive impairment	–	–	+	+	+	+	+	+
Cutaneous findings	–	–	–	–	–	–	–	–
Ocular findings	NA	NA	NA	NA	NA	NA	Probable cataract	NA
Microcephaly and facial Dysmorphism	–	–	+	+	+	+	+	–
Epilepsy	–	–	–	–	–	–	–	–
MRI	Mild cortical atrophy	NA	Normal	NA	NA	NA	CC atrophy; PWMA; mild cortical atrophy	NA

**Table:** 

**Family N°/origin**	**Case/Denmark** **(****22****)**	**HSP190/Japan** **(****3****)**		**HSP48/Japan** **(****3****)**		**Case/Italian** **(****23****)**		**Case4/ China** **(****24****)**	**Case/ Hungary** **(****25****)**
ALDH18A1 variants	c.1741G>A, p.E581K; c.251G>A, p.R84Q	c.1321C>T, p.R441^*^; c.1994G>A, p.R665Q		c.383G>A, p.R128H (hom)		c.1112G>A, p.R371Q; c.1490G>A, p.S497N		c.725G>A, p.S242N (hom)	c.-28-2A>G; c.383G>A, p.R128H
Individual N° (sex)	III-3(M)	II-1(M)	II-2(M)	II-1(M)	II-4(F)	II-1(M)	II-2(M)	IV-1/F	F
Age at onset (years)	1.5	<6	<6	32	NA	1	3	10	<1
Symptoms at onset	Toe walking	Spastic gait	Spastic gait	Spastic gait	NA	Developmental delay	Gait disturbances	Gait disturbances	Tremor
Disease duration (years)	17.5				NA	40	35	22	5
Spasticity at gait	+	+	+	+	NA	+	+	+	+
Weakness	NA	NA	NA	NA	NA	+	+	+	NA
Increased reflexes LL	NA	NA	NA	NA	NA	+	+	+	NA
Increased reflexes UL	NA	NA	NA	NA	NA	+	+	–	NA
Extensor plantar response	NA	NA	NA	NA	NA	+	+	+	NA
Hoffman sign	NA	NA	NA	NA	NA	+	+	+	NA
Decreased vibration sense at ankles	NA	NA	NA	NA	NA	+	+	NA	NA
Urinary symptoms	NA	NA	NA	NA	NA	NA	NA	NA	NA
Cerebellar signs	Tremor	+	+	–	NA	NA	NA	NA	Tremor
Cognitive impairment	+	+	+	+	NA	+	+	–	+
Cutaneous findings	–	–	–	–	NA	–	–	–	–
Ocular findings	NA	NA	NA	NA	NA	NA	NA	NA	–
Microcephaly and facial dysmorphism	–	–	–	–	NA	+	+	–	+
Epilepsy	+	–	–	–	NA	–	+	–	+
MRI	Normal	Mild cerebellar atrophy	Mild cerebellar atrophy	Normal	NA	Increase in the prominence of the cortical sulci	NA	Normal	CC hypoplasia; thin white matter

*ID, intellectual delay; MD, motor delay; PMD, psychomotor delay; GR, growth retardation; LL, lower limbs; UL, upper limbs; CC, corpus callosum; PWMA, periventricular white matter anomalies; NA, not available. Cerebellar signs include dysdiadokinesia, ataxia, nystagmus, intention tremor, speech-slurred, or scanning, hypotonia*.

### Molecular Studies

We sequenced the exomes of the proband and her parents to 100X average depth in target regions, with 96% of the target base pairs on average having at least 20X coverage. WES analysis identified a heterozygous missense variant in *ALDH18A1* (chr10: 95628421A>G hg38, NM_002860: exon8: c.880T>C: p.S294P), which was inherited from her mother (Figure 1B). This variant was completely absent from the ExAC, 1,000 Genomes Project, and Genome Aggregation Database (gnomAD) genome and exome databases. It also showed high evolutionary conservation (GERP score: 5.79), was predicted to be deleterious by at least three different algorithms (PolyPhen-2, MutationTaster, CADD), and was found to be located in the G5K domain of P5CS. Based on these results, we classified the variant as “Likely Pathogenic” according to ACMG/AMP guidelines (PM1, PM2, PM3, and PP1) (26).

As the clinical presentation of the proband was compatible with spastic paraplegia, and we did not find any other rare candidate variants in either the exon or conventional splicing region in the exome sequence, we then re-examined the exome data and found a potentially deleterious variant in the intron 1. This variant (chr10: 95653418T>C hg38, NM_002860: c.-28-13A>G) was intronic, completely absent from ExAC and 1,000 Genomes Project, occurred at extremely low frequencies among East Asian populations according to the gnomAD database (1/5,196), and has not been previously reported. This variant was inherited from her father and segregation in the family was compatible with pathogenicity of the variant. *In silico* analysis using SpliceAI, a novel tool for cryptic splice site prediction based on artificial neural network deep learning (15), predicted the disruption of an acceptor site located 13 bp downstream of the c.-28-13A>G variant. The loss of the original acceptor splice site likely led to the transcriptional skipping of exon 2.

We therefore sought to validate the SpliceAI prediction through analysis of cDNA reverse transcribed from RNA extracted from the peripheral blood samples of patients and family members. Two transcripts were observed in gel electrophoresis and Sanger sequencing confirmed the prediction that exon 2 was absent in the truncated transcript due to transcriptional skipping caused by the c.-28-13A>G variant (Figure 1C). The percentage of truncated transcript was 14%, which was quantified using HTS (Supplementary Table 2). This novel mRNA isoform lacked the original start codon necessary for initiation of full length *ALDH18A1* transcription, instead initiating mRNA transcription at the second possible start codon, in a different downstream reading frame (Figure 1D). Each of these reading frames, i.e., for the correct and aberrant start codons, resulted in a different amino acid sequence, and hence one of which was non-functional P5CS protein.

### Functional Analysis of *ALDH18A1* Mutations

The P5CS enzyme is known to catalyze proline and ornithine biosynthesis. The effects of disease-related mutations in *ALDH18A1* are correlated with loss or decrease of P5CS function (2, 24, 27). To validate the functional involvement of *ALDH18A1* in SPG9 in this family, we next performed ELISA analysis of the proband's plasma. The results showed that plasma P5CS concentration in patient II-2 was significantly decreased compared to that of the three, gender matched healthy controls (*p* < 0.05) (Figure 1E). Based on this functional validation, we proposed that compound heterozygosity for these two mutations most likely explains the phenotype of the patient.

## Discussion

It has been estimated that genetic variants that affect splicing comprise roughly 9–10% of the pathogenic variants in rare genetic disorders (15, 28, 29), although this number appears very likely to be an underestimation. To date, disease-causing intronic splicing mutations have been reported in only a few genetic disorders such as ataxia-telangiectasia and neurofibromatosis type I (30, 31). Recently, a deep intronic splice mutation causing SPG7 was identified through detection by WGS, which led to a diagnosis of HSP (9). This discovery shed light on the importance of studying mutations at both the DNA and RNA levels. A number of *in silico* prediction tools have been developed to assess the effects of DNA sequence variations on splicing, which have substantially improved diagnosis of several human genetic diseases (32). In this work, the pathogenicity of intronic splice mutation was predicted through Illumina SpliceAI (15). Based on a deep learning neural network, SpliceAI can predict splice junctions from random pre-mRNA transcript sequences and generate anticipated start and end positions of aberrant exons with very high accuracy. The SpliceAI-predicted Delta scores and the position of the lost accepter for the variant (-28-13A>G) were 0.41 and −13, respectively, which indicated that exon2 was skipped (Figures 1C,D). The predicted length of the aberrant exon matched its observed length in the patient-derived cDNA sequence data.

In this study, we report an SPG9B family in which WES coupled with cDNA sequencing led to the identification of causative mutations. To our knowledge, this is the first description of a hidden intronic mutation in *ALDH18A1*, outside the essential GT and AG splice site dinucleotides, and moreover, only eight families with bi-allelic mutations in *ALDH18A1* have been described to date. Clinical details for these patients and the previously reported SPG9B patients are presented in Table 1. Mutations in *ALDH18A1* were initially identified in autosomal recessive or dominant neurocutaneous syndrome, characterized by severe developmental delay with marked cognitive impairment, associated with progeroid features, cutis laxa, joint hyperlaxity, short stature, cataract, and frequent microcephaly (2, 33, 34). Pyramidal signs have been found in patients with neurocutaneous syndrome, although not cardinal. Subsequently, it became clear that *ALDH18A1* was the causal gene in some families with autosomal dominant or recessive HSP (SPG9A and SPG9B) (2). Pyramidal syndrome was reported as severe in SPG9 patients, while cutis laxa has never been noted, although it was previously considered to be an obligate sign of neurocutaneous syndrome (Table 1). Moreover, the clinical features of patients with SPG9B largely differ from those with SPG9A: they generally exhibit earlier onset and higher severity than SPG9A patients (27). In addition, unlike SPG9A, SPG9B patients share common features with those previously reported for neurocutaneous syndromes: developmental delay (7/14), intellectual deficiency (13/14), short stature (7/14), and facial dysmorphism (9/14) (2). Interestingly, these syndromes were demonstrated in 9/10 Caucasian patients, but none of which were observed in our patients. Notably, the clinical presentation was not homogeneous among the respective families, regardless of differences in their ethnicity. Age, disease duration, lifestyle, and genetic differences among ethnic groups are all potential modifiers underlying this phenotypic variability.

A spectrum of 13 mutations in the *ALDH18A1* gene have been identified in 17 patients with autosomal recessive HSP, including 10 missenses, 2 splicing mutations, and a non-sense mutation (Table 1). Most notably, the previously reported p.R128H mutation was found in seven patients (2, 3, 25), making it the most frequently occurring *ALDH18A1* mutation thus far identified in SPG9B patients. The patients reported to carry the p.R128H mutation invariably exhibited an earlier age of onset. However, truncating mutations, unlike other mutation types, have not been associated with an early age at onset or severe presentation. Moreover, genotype–phenotype correlations have been difficult to establish due to the complexity of both the phenotype and the function of the gene. Variability in the age at onset ranges from <1 to 32 years in SPG9B patients and no significant correlation has been established between phenotype and mutational class. It is therefore possible that other genotype–phenotype correlations will emerge as larger numbers of patients are studied.

## Conclusion

With this report, we expand the genotype and phenotype spectrum of SPG9B and provide further evidence that RNA splicing assays, in conjunction with clinical and family inheritance analyses, can determine the clinical significance of intronic variants. In addition, the identification of this splicing mutation within intronic sequence should prompt closer scrutiny of *ALDH18A1* non-coding genomic sequence in HSP patients for whom coding mutations in HSP-causative genes have not been identified.

## Data Availability Statement

The datasets presented in this study can be found in online repositories. The names of the repository/repositories and accession numbers can be found at: NCBI GenBank; PRJNA699107 and PRJNA699100.

## Ethics Statement

The studies involving human participants were reviewed and approved by the First Affiliated Hospital of Fujian Medical University. The patients/participants provided their written informed consent to participate in this study.

## Author Contributions

Conception and drafting of the work were performed by Y-JC and XL. Data acquisition and data interpretation were performed by Y-JC, Z-QZ, M-WW, Y-SQ, R-YY, E-LD, ZZ, and H-TZ. Revision of the manuscript for intellectual content was conducted by XL, NW, and W-JC. All authors have read and approved the final manuscript.

## Conflict of Interest

The authors declare that the research was conducted in the absence of any commercial or financial relationships that could be construed as a potential conflict of interest.
