# Supplementary material for: Novel Compound Missense and Intronic Splicing Mutation in ALDH18A1 Causes Autosomal Recessive Spastic Paraplegia
Source: Front Neurol. 2021 May 19;12:627531. doi: 10.3389/fneur.2021.627531 (PMC8170465; doi:10.3389/fneur.2021.627531)
Supplement: Supplementary file 1 [file Data_Sheet_1.docx]

Supplementary Figure 1


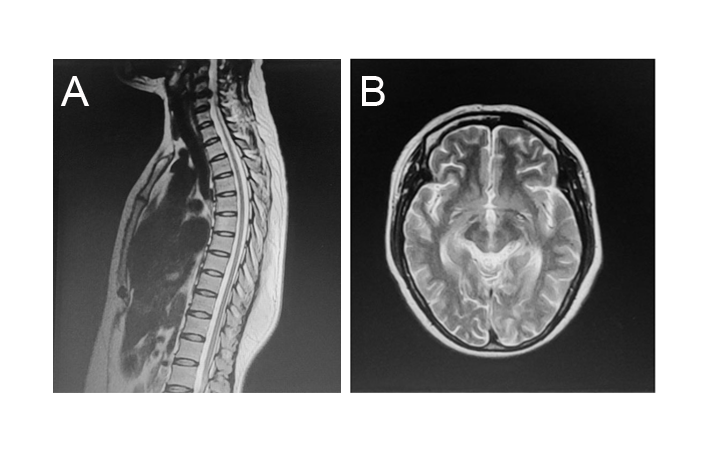


Fig. S1 The spinal and brain MRI of proband. The spinal MRI presented atrophy of thoracic spinal cord (A). The brain MRI was normal (B).

Supplementary Table 1. Plasma amino-acid levels in the proband.

|  | Plasma level (μM) | Normal range (μM) |
| --- | --- | --- |
| Proline | 814.45 | 250-2500 |
| Arginine | 21.57 | 1-70 |
| Ornithine | 19.27 | 7-120 |
| Citrulline | 20.52 | 5.5-45 |

Supplementary Table 2. The reads number of ALDH18A1 transcripts of the cases carrying c.-28-13A>G.

| Sample | Total-reads-number | Full-length-reads-number | Percentage of truncated transcript |
| --- | --- | --- | --- |
| II-2 | 1994727 | 1710367 | 14% |
| II-1 | 1780199 | 1583484 | 11% |
